# Principles of Molecular Oncology: Second Edition

**DOI:** 10.1038/sj.bjc.6601866

**Published:** 2004-10-26

**Authors:** H Laman

**Affiliations:** 1 CRC Viral Oncology Group, Wolfson Institute for Biomedical Research, Bruciform Building, UCL Gower Street, University College London, UK

This second edition of *Principles of Molecular Oncology* comes just 4 years after the first, and considering that we are now in the ‘postgenomics’ era, it does well to keep pace with the advances of this rapidly evolving field. The text catalogues an amazing array of molecular biology-inspired techniques and approaches for the diagnosis, treatment, and understanding of cancer, which is even more astounding when weighed against the fact that when DNA was discovered 50 years ago, ‘cancer knowledge was based on epidemiology and pathology, and treatment consisted of surgery and radiation therapy.’ This text offers the reader a comprehensive overview of how 25 years of molecular biology have been brought to bear on the problems of this complex disease.

The opening chapter introduces the current state of affairs in cancer biology, including its epidemiology, diagnosis and treatment, the ‘older’ and ‘newer’ hypotheses surrounding its causes, and the contributions of diet, viruses, nicotine/tobacco, and hereditary predisposition to its development. Despite the vast increase in our knowledge, the existing therapies have had a relatively minor effect on mortality. As such, the anticipated dawn of the ‘era of targeted therapies' is eagerly awaited by all.

One kind of targeted or tailored approach is addressed in the first section of the book, where molecular markers of cancers are discussed. Realising that earlier detection is a major advantage in cancer treatment, scientists are searching for more and better markers to be used as both diagnostic and prognostic factors to tailor the utility and efficacy of treatments. The identification of markers in sporadic and heritable tumours, and in solid tumours is discussed. Also, the hunt for circulating tumour markers to assess tumour burden is detailed. Traditional approaches of histological diagnosis and karyotyping are presented side by side with the newer approaches of gene expression and protein profiling.

The middle section of the book explains our molecular and mechanistic understanding of the deregulated cellular pathways in cancer development, including signal transduction pathways, cell proliferation, angiogenesis, invasion/metastasis, drug resistance, and genomic instability. As these are each robust areas of research, there is a large amount of information here, and each of the chapters is comprehensive and detailed in its dissection of these recognised abnormalities of cancerous cells. Although some chapters are more readable than others, in general the information presented is concise and points the interested reader towards the primary sources of information.

The final section deals with the therapies arising from molecular biology. As researchers try to benefit from all this new information, the hope is to move on from ‘scientifically interesting to clinically useful.’ Although the approaches being taking are research-driven, spanning suicide gene therapy, monoclonal antibody therapies, and assisted drug-design and development, understanding the function of a gene does not necessarily mean it will be a good target for cancer treatment. This is a major deficiency facing molecular oncologists today: with so many possible genes, which represent the best, ‘druggable’ target on which to focus our energies. Nevertheless, a trickle of next-generation drugs, whose mechanisms of action are understood, are emerging. These successes are hailed repeatedly and inspire the hope that pervades this textbook and the field in general.

Despite the fact that there are 65 contributors to this edition of *Principles* from Europe and America, it is highly readable, with most of the chapters being well written and up-to-date, even listing the newest compounds and their ongoing clinical trials. Each chapter is well referenced, although the text is not very well indexed, making it difficult to find particular genes or drugs of interest within the chapters. As there are many authors, the book is repetitive in places, but this only serves to underscore the fundamental importance of certain pathways, like p53, in cancer. Overall, this is a thorough treatment of a fast-moving and exciting field. As such, it will appeal to clinicians and scientists, and it should spur on young medical students or budding researchers to the coming era of molecular approaches to human diseases.

